# Host biology and genomic properties of Plumeria mosaic virus, a tobamovirus discovered in a temple tree in India co-infecting with frangipani mosaic virus

**DOI:** 10.3389/fmicb.2022.1030042

**Published:** 2022-11-02

**Authors:** Alok Kumar, Vikas Solanki, Akshay Katiyar, Bikash Mandal

**Affiliations:** ^1^Advanced Centre for Plant Virology, Division of Plant Pathology, Indian Agricultural Research Institute, New Delhi, India; ^2^School of Plant Sciences, College of Agriculture and Environmental Sciences, Haramaya University, Dire Dawa, Ethiopia

**Keywords:** Plumeria mosaic virus, tobamovirus, RT-PCR, frangipani mosaic virus, temple tree, differential host, complete genome

## Abstract

Temple tree (*Plumeria rubra* f. *acutifolia*), an important fragrant-flower tree extensively used in the urban landscaping is known to be infected with a tobamovirus, frangipani mosaic virus (FrMV). In this study, we describe another tobamovirus, Plumeria mosaic virus (PluMV) infecting temple tree in India. PluMV was isolated from an old temple tree co-infected with FrMV. The presence of another tobamovirus was initially realized based on the distinct symptoms on *Gomphrena globosa* (globe amaranth), a non-host of FrMV. PluMV was highly transmissible through simple rub-inoculation. In host-range study, brinjal (*Solanum melongena*), chilli (*Capsicum annuum*)*,* datura (*Datura stramonium*)*,* globe amaranth and tobacco (*Nicotiana benthamiana, N. glutinosa, N. tabacum* cv. Xanthi) could differentiate PluMV from FrMV. The complete genome sequence of PluMV was determined (6,688 nucleotides [nt], GenBank KJ395757), which showed the genome structure typical of tobamovirus encoding four proteins: small replicase (3,549 nt/130 kDa), large replicase (5,061 nt/188 kDa), movement protein (770 nt/29 kDa) and coat protein (527 nt/19 kDa). The 5′ and 3′ UTR of PluMV contained 91 and 284 nt, respectively. The PluMV genome was 45 nts longer than that of FrMV and shared only 71.4–71.6% sequence identity with FrMV and  < 50% sequence identity with the rest of the other members of the genus *Tobamovirus*. PluMV shared a close but a divergent evolutionary relationship with FrMV. Based on the species demarcation guidelines of ICTV (<90% genome sequence identity), PluMV was considered as a new tobamovirus species. As PluMV was serologically related with FrMV, differential diagnostic assays such as simplex and duplex RT-PCR were developed, which revealed that PluMV naturally existed in both the species of temple tree, *P. rubra* f. *acutifolia* and *P. rubra* f. *obtusa* in India either alone or in mixed infection with FrMV.

## Introduction

The members of the genus *Tobamovirus* (family *Virgaviridae*) have rod-shaped virions of about 300 × 18 nm size and ssRNA genome of ~6.3 – 6.6 kb. Tobacco mosaic virus is the first member discovered under the genus *Tobamovirus*. Subsequently, as many as 37 confirmed and 2 tentative tobamovirus species have been reported infecting several plant species (https://ictv.global/taxonomy/). Tobamoviruses are highly stable and contagious that spread through direct contact with the infected plant materials and/or contaminated soil/water, but not by any insect-vector with specific biological specificity (Adams et al., 2009). The genome of tobamoviruses consists of four open reading frames (ORFs) that encode four proteins. The ORF1 and ORF2 encode smaller (124–132 kDa) and larger (181–189 kDa) replicase proteins (Rep), which are expressed directly from the genomic RNA and help in viral replication (Lewandowski and Dawson, 2000). The ORF3 and ORF4 encode movement protein (MP) and coat protein (CP), which are translated from the sub-genomic RNAs (Ikea et al., 1993), and are responsible for cellular movement and virion formation, respectively (Ikea et al., 1993). Tobamoviruses are classified into three subgroups considering the difference in infected plant species and genome architecture. The subgroup-I includes the members those infect solanaceous crops and the MP and CP genes in the genome is not arranged in overlapping manner. The subgroup-II includes the members those infect legumes, cucurbits, and some other crops, and their MP and CP genes are arranged with slightly overlapping manner in the genome. The subgroup III includes the members that infect brassicas, asterids and some other plant species, and their MP and CP genes are overlapping in a greater extent compared to those in the subgroup II (Pagan et al., 2010; Wylie et al., 2013).

The temple tree or frangipani (*Plumeria* sp.*,* family Apocynaceae) is a deciduous ornamental plant, widely grown for its beautiful foliage and fragrant flowers, and extensively used in urban landscaping. It also has medicinal property, and is used for treating skin inflammation, indigestion, high blood pressure, hemophilia, cough, dysentery, and fever (Bihani et al., 2021). The temple tree, native to Mexico, Central America, Colombia, and Venezuela is commonly grown in the tropical and subtropical regions of the World including India. The temple tree was known to be infected by a tobamovirus, frangipani mosaic virus (FrMV) in Australia (Francki et al., 1971) and India (Varma and Gibbs, 1978) initially based on their host-reactions and virions morphology, and subsequently, the virus was identified as a distinct tobamovirus species based on the genome sequence information generated for the isolate from China (Deng et al., 2000; Lim et al., 2010) and India (Kumar et al., 2015).

While studying the occurrence of FrMV in temple trees at the campus of Indian Agricultural Research Institute (IARI), New Delhi, an old temple tree (*P. rubra* f. *acutifolia*) (>35 years) was observed to exhibit varieties of symptoms such as mosaic, bronzing, vein banding, necrotic spots, and ring-spots on leaves. The RT-PCR test showed the presence of FrMV in this tree. The inoculum prepared from this tree when was used to inoculate different plant species, *Gomphrena globosa* developed bright red local lesion symptoms that was strikingly different from FrMV as *G. globosa* was found to be a non-host of FrMV in our previous study (Kumar et al., 2015). This prompted us to investigate the virus isolate obtained through *G. globosa*, which led to the discovery of a novel tobamovirus, Plumeria mosaic virus from a temple tree, mixed infected with FrMV. The preliminary report of PluMV was presented in an International Conference of Indian Virological Society (Kumar et al., 2013). In this paper, we systematically describe the isolation of the new virus culture, host biology, complete genome sequence and evolutionary relationships of PluMV with FrMV and other members of the genus *Tobamovirus.* Further, simplex and duplex RT-PCR based diagnostic assays were developed for differentiating PluMV from FrMV, which were successfully utilized for demonstrating the natural existence of PluMV in temple trees independently or as a mixed infection with FrMV.

## Materials and methods

### Virus source and host biology study

The symptomatic leaf sample from an old temple tree (*P. rubra* f. *acutifolia*) at IARI campus was collected during 2010 and analyzed by leaf dip electron microscopy (EM), RT-PCR, and sap-transmission. EM was conducted using a small disc of leaf following the methods described by Hilchborn and Hills (1965). The grid was stained with uranyl acetate (2%), washed with distilled water, air dried, and analysed in a transmission electron microscope (Model JEOL 100 CX-II). RT-PCR was conducted using primers specific to CP genes of FrMV (Kumar et al., 2015). Sap-inoculation was conducted using the leaf extract prepared by grinding the leaf sample in 0.1 M phosphate buffer, pH 7.2 at the ration of 1:5. The test plants were pre-dusted with Carborundum powder (320 grit) and inoculated with the extracted sap. The plants were allowed to grow at 30–35°C in the greenhouse and were observed for symptom expression.

**Figure 1:**
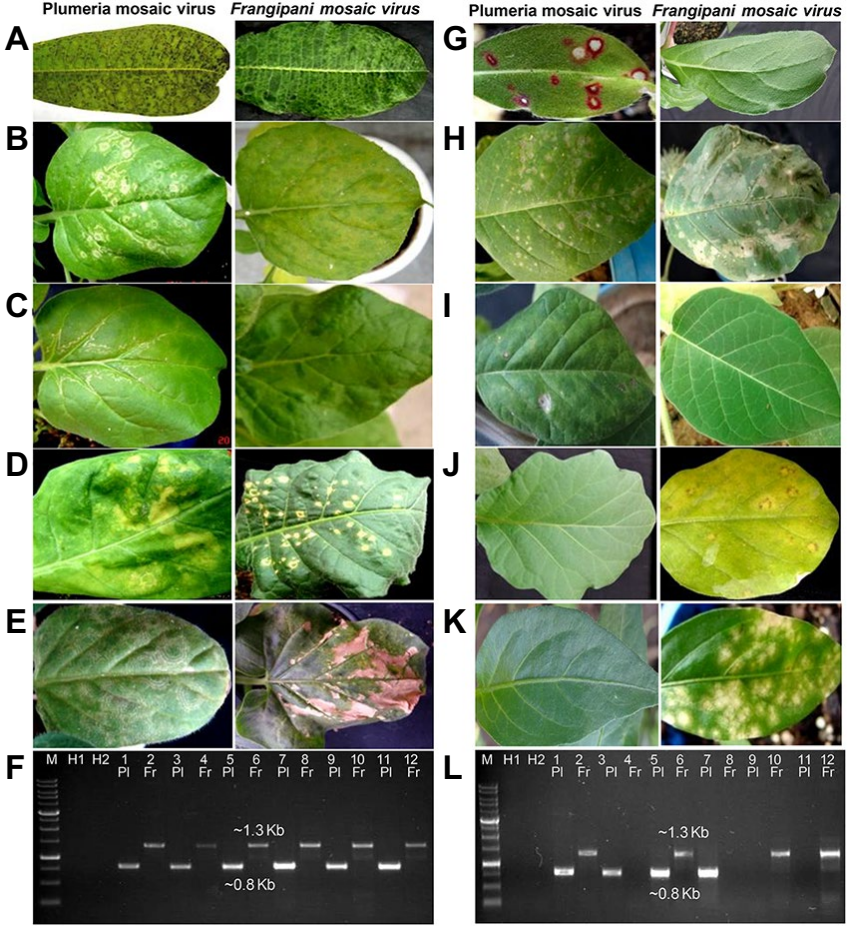
Comparison of symptoms of plumeria mosaic virus (PluMV-Plu-Ind-1) and frangipani mosaic virus (FrMV-Ind-1) on various hosts following sap inoculation. **(A)** *Plumeria rubra* f. *acutifolia*, **(B)** *Nicotiana benthamiana* local leaf, **(C)** *N. benthamiana* systemic leaf, **(D)** *N. tabacum* cv. Xanthi, **(E)** *N. glutinosa*, **(G)** *Gomphrena globosa*, **(H)** *Datura stramonium* local leaf, **(H,I)** *Datura stramonium* systemic leaf, **(J)** *Solanum melongena*, **(K)** *Capsicum annuum*. (F & L) Confirmation of transmission of the virus to these hosts by RT-PCR. **(F)** Lane 3–4: *P. acutifolia*; Lane 5–6: local *N. benthamiana*; Lane 7–8: systemic *N. benthamiana*; Lane 9–10: *N. tabacum*; Lane 11–12: *N. glutinosa*. **(L)** Lane 3–4: *G. globosa*; Lane 5–6: local *D. stramonium*; Lane 7–8: Systemic *D. stramonium*; Lane 9–10: *S. melongena*; Lane 11–12: *C. annuum*. Pl: PluMV; Fr: FrMV. M: Marker; H1 and H2: Healthy control; Lane 1: PluMV +ve control (Cloned DNA); Lane 2: FrMV +ve control (Cloned DNA).

The virus isolate obtained from a single local lesion on *G. globosa* was designated as, PluMV-Plu-Ind-1 and maintained on *N. benthamiana.* The FrMV-Ind-1 culture was established from a single local lesion on *C. annuum* and was maintained on *N. benthamiana* in a separate greenhouse. To compare the host reactions of PluMV-Plu-Ind-1 and FrMV-Ind-1, the virus isolates were used separately for the sap-inoculation to the different plant species (Figure 1; Table 1) at 3–5 leaf stage and maintained in the two separate greenhouses. The symptom expression was recorded and the association of the virus was confirmed by direct antigen coated enzyme-linked immunosorbent assay (DAC-ELISA) using antiserum developed from purified virus preparations of FrMV, and RT-PCR using CP-gene specific primers to FrMV, developed in our previous study (Kumar et al., 2015). All the plant samples were tested again by RT-PCR at the later stage, using the primers specific to each virus, developed in this study (Table 2).

**Table 1:** Comparison of local and systemic symptoms of plumeria mosaic virus isolate, PluMV-Plu-Ind-1 and frangipani mosaic virus isolate, FrMV-Ind-1 on different plant species.

Hosts	PluMV-Plu-Ind-1 symptoms		FrMV-Ind-1 symptoms	
	Local	Systemic	Local	Systemic
*Plumeria rubra* f. *acutifolia*	NS	Brown mosaic	NS	Greenish mosaic, chocolate spots and necrotic ring with central spots
*Plumeria rubra* f. *obtusa*	NS	Yellow mosaic with brown necrotic spots	NS	Greenish mosaic with necrotic spots
*Nicotiana benthamiana*	Whitish ring-spot	Whitish wavy lines, mosaic mottling and blistering	Chlorotic spots	Mosaic, mottling and blistering
*N. tabacum* cv. Xanthi	Whitish ring-spots and mottling	NS	Necrotic white lesions	NS
*N. glutinosa*	Concentric whitish ring	NS	Large necrotic lesions	NS
*Gomphrena globosa*	Red spots	NS	NS	NS
*Datura stramonium*	Chlorotic spots	Chlorosis with mottling	Large blighted patches	NS
*Solanum melongena*	NS	NS	Chlorotic spots	NS
*Capsicum annuum*	NS	NS	Chlorotic lesions	NS

NS: No symptoms.

**Table 2:** List of the primers used for the amplification of the complete genome of plumeria mosaic virus (PluMV) from *Plumeria rubra* F. *acutifolia,* and for the detection of PluMV and frangipani mosaic virus (FrMV).

Primer^a^ name	Primer sequence (5′ to 3′)	Primer location (nt)	Annealing temperature (°C)	Amplicon length (~Kb)	Remarks
*For the amplification of complete genome of PluMV*					
BM116R	tgacaagtcgacttgtcatatttagaaacatcaagctc	4,348–4,373	58	1.3	Part of rep
BM115F_d_ ^b^	gtawktttwmawywwttwmyaaywacaacaa	1–31			
BM204R	caatgacttggtcaaagtcctca	3,231–3,253	58	3.1	5′ UTR and part of rep gene
BM239F	ggatcc ccaaagggtaatatttaccaacaatt	1–26	58	1.9	5′ UTR and part of rep gene
BM222R	tcgcagccaatgcactctccc	1967–1987			
BM348F	gctagcaaaacatggcttttgac	2,455–2,477	58	3.0	part of replicase and movement protein genes
BM240R	gtcgac ctaaatatcttcattatctccacttt	5,793–5,818			
BM649F	aattacttcccaagtcgatgactag	5,121–5,145	58	1.3	part of movement protein and coat protein genes
BM140R	gcgtaa gtcgac ttacgcggtagtagtacccg	6,382–6,404			
BM205F	gatgcttcggggttggtatggg	6,321–6,342	58	0.4	part of coat protein and 3′ UTR
BM119R	agcccagtcgactgggccgctaccgggggtta	6,668–6,686			
*Primers used for 5′ RACE (5′-Full RACE core set)*					
BM667R	(p)-aacaaaaagtatcaaccaaag	1,075	42	1.1	cDNA synthesis
BM520F	ggcaggcttacatcgtttttcga	579–601	62	1.0	Outer RACE
BM530R	actctggcaatatctctaatgtcc	478–500			
BM244F	aagatggtagttacgccgtcg	704–724	62	0.8	Inner RACE
BM452R	ttgcaacaatgaacatacgagcgt	444–467			
*Primers used for 3′ RACE*					
BM210(Adaptor)	gcgagcacagaattaatacgactcactataggttttttttttttvn	6,688	42	Full length	cDNA synthesis
BM649F	aattacttcccaagtcgatgactag	5,121–5,145	58	1.6	Outer RACE
Outer RACE primer	gcgagcacagaattaatacgact	Outer part of adaptor			
BM205F	gatgcttcggggttggtatggg	6,289–6,310	58	0.4	Inner RACE
Inner RACE primer	cgcggatccgaattaatacgactcactatagg	Inner part of adaptor			
*Primers used for the specific detection of plumeria mosaic virus and frangipani mosaic virus*					
BM348F	gctacg aaaacatggcttttgac	2,455–2,477	58	0.8 Kb	PluMV
BM204R	caatgacttggtcaaagtcctca	3,231–3,253			
BM520F	ggcaggcttacatcgtttttcga	579–601	58	1.2 Kb	
BM521R	aaacaagcgcctacgttaacctt	1744–1766			
BM200R	aattcctgttttgaacttagattcg	4,282–4,306	58	2.0 Kb	FrMV
BM523F^c^	gacggcaaccttgaacaatttgc	2,333–2,355			
BM607R	attgtagttgcatcaaaattattaagta	3,637–3,664	58	1.3 Kb	

a Position of the primers on the viral genome are shown in Figure 1,

b BM115_d_F is a common degenerate primer with BM116R and BM204R.

c BM523F is common primer with BM200R and BM607R,

F: Forward, R: Reverse, underline: Restriction site.

### RT-PCR and cloning of genome fragment

**Figure 2:**
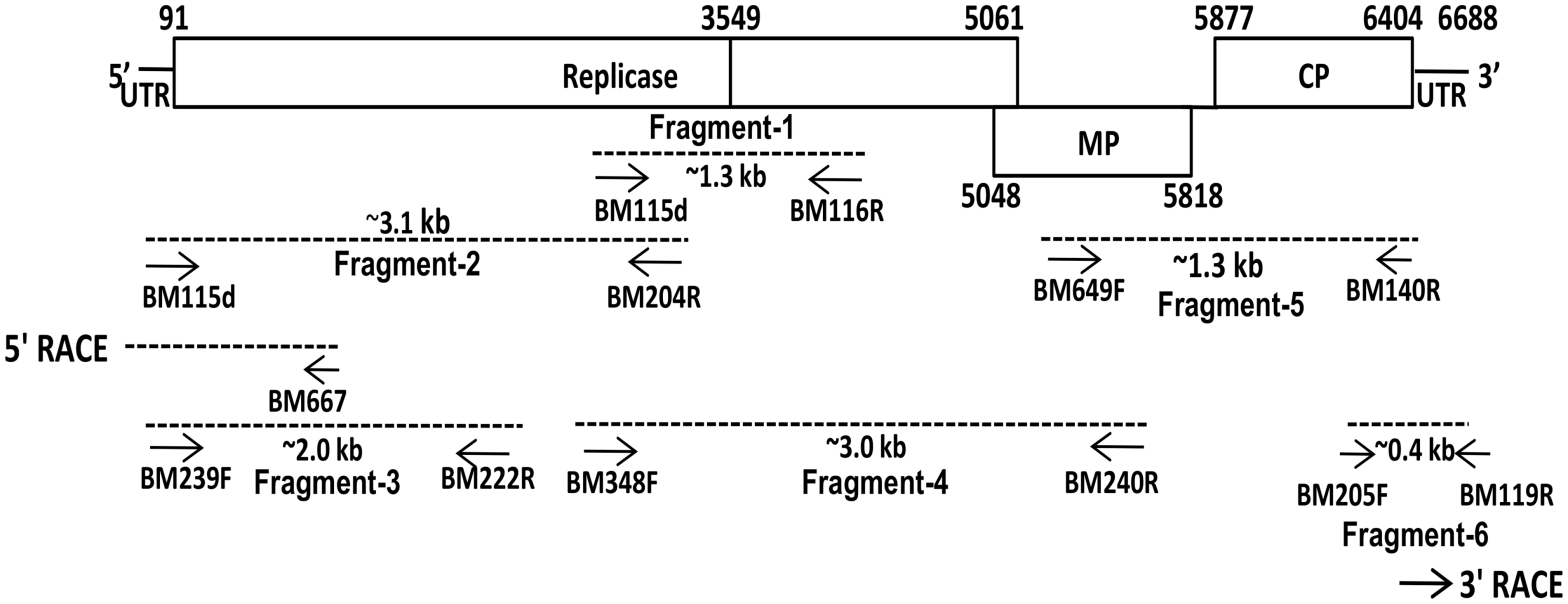
Schematic presentation of the strategy used for the amplification of complete genome of plumeria mosaic virus (pluMV-Plu-Ind-1 isolate) infecting *Plumeria rubra* f. *acutifolia*. Small arrows indicate the positions of various primers on the genome. The genome segments, which were used for sequencing, are indicated as dotted lines.

The RNA was extracted from the symptomatic leaves of *G. globosa* showing red chlorotic lesions using RNeasy Plant Mini Kit (Qiagen. Inc. Chatsworth, CA). The viral genome was amplified by RT-PCR in six different overlapping fragments that covered entire viral genome (Table 2; Figure 2). Further, both the terminal fragments containing 5′- and 3′-UTR were amplified using 5′-Full RACE core set kit (Takara, Shiga, Japan) and FirstChoice RLM-RACE kit (Thermo Fischer Scientific, USA), respectively (Table 2; Figure 2). Initially, the primers were prepared based on the genome sequences of FrMV-P (HM026454), FrMV-Ind-1 (JN555602), and other tobamoviruses. Subsequently, primers were designed based on the genome sequences generated in this study.

For preparing cDNA, the reaction mixture contained 5x First-Strand buffer (4.0 μl), 10 mM dNTP mix (1.0 μl), 20 mM DTT (1.0 μl), 10.0 μM reverse primer (2.0 μl), 100 Units/μl SMARTScribe™ reverse transcriptase enzyme (Clontech, USA) (1.0 μl), and RNA template (400–500 ng, 10 μl). The final volume was adjusted to 20.0 μl with nuclease free water. The reaction mixture was allowed for 90 min at 42°C followed by inactivated at 70°C for 15 min in the Biometra *T* Personal thermal cycler.

The cDNA (2.0 μl) was used for PCR with 10x Ex Taq buffer (5.0 μl), 2.5 mM dNTPs (4.0 μl), 10 μM of each primer (2.0 μl), and 1.25 U of Ex-Taq DNA polymerase (Takara, Shiga, Japan). The final reaction mixture was adjusted to a volume of 50 μl with the nuclease-free water. The amplification conditions were: 40 cycles, each having denaturation at 98°C for 10 s, annealing at 58–62°C (Table 2) for 45 s, and extension at 72°C for 1.0 min/kb. The final extension was allowed at 72°C for 10 min. The amplified products were resolved in agarose gel, purified using gel purification kit (Macherey-Nagel, Germany), and cloned in pT&A vector (Real Biotech Corporation, Banqiao, Taiwan) using the manufactures’ protocol.

### Genome sequence analysis

All the cloned fragments of the viral genome were sequenced from both the ends using the commercial facility at Chromous biotech (Bengaluru, India). The vector sequences from all the clones were removed, and assembled to obtain the complete genome sequence of the virus isolate, PluMV-Plu-Ind-1. The ORF finder software available at NCBI site (http://www.ncbi.nlm.nih.gov/gorf/gorf.html) was used to determine the coding sequences. The sequence was compared using basic local alignment search tool (http://www.ncbi.nlm.nih.gov /blast) and BioEdit software (Hall, 1999). The phylogenetic and molecular evolutionary analyses were conducted based on the amino acid sequences of each ORF of PluMV-Plu-Ind-1, and the corresponding sequence of other tobamovirus isolates using the maximum likelihood method in the MEGA version 11 (Tamura et al., 2021) with 1,000 bootstrap values.

### Simplex and duplex RT-PCR

Two pairs of primers, specific to each virus, were designed from the Rep gene sequence of PluMV-Plu-Ind-1 and FrMV-Ind-1, for developing the RT-PCR based diagnostic technique specific to each of these viruses (Table 2). The conditions for the RT-PCR using these primers were optimized using the respective cloned DNA fragments (Figure 2). The duplex PCR conditions were optimised for the primer pairs, BM348F/BM204R and BM523F/BM607R using the mixture of the respective cloned DNA of each virus. Both the simplex and duplex RT-PCR were validated using leaf samples from inoculated plants. Further, duplex RT-PCR was performed using leaf samples collected from fields to confirm the existence of PluMV in other temple trees.

## Results

### Isolation of PluMV

The EM analysis of leaf sample from an old temple tree plant that exhibited mosaic, vein banding, bronzing, ring-spot and necrotic spot symptoms revealed the presence of numerous rod-shaped virions (300 × 18 nm) similar to tobamovirus. The sap-transmission from the leaf sample of the above temple tree resulted in development of local lesion symptoms in *G. globosa, C. annuum,* and *S. melongena*. The subsequent transmission of the virus from the local lesion hosts, *C. annuum,* and *S. melongena* to *N. benthamiana* resulted in the development of similar type of mosaic, mottling, and blistering symptoms. However, transmission from the local lesion tissues of *G. globosa* resulted in different symptoms such as whitish ring-spots on the inoculated leaves, and mosaic, mottling, and whitish wavy lines on systemic leaves of *N. benthamiana* (Figure 1). Further, the virus isolate from *G. globosa* showed weak amplification in the RT-PCR with the CP gene specific primers to FrMV compared to the virus isolates from *C. annuum* and *S. melongena*. Due to the difference in symptomatology and amplification of CP gene, the virus isolate obtained from *G. globosa* was designated as PluMV-Plu-Ind-1. The virus transmission and RT-PCR results indicated that there was a mixed-infection of viruses/strains in the temple tree.

### Comparison of host-reactions of PluMV-Plu-Ind-1 with FrMV-Ind-1

The comparison of host-reactions following mechanical sap inoculation to the various plant species with PluMV-Plu-Ind-1 and FrMV-Ind-1 is presented in Figure 1; Table 1. PluMV-Plu-Ind-1 caused brown mosaic symptoms on *P. rubra* f. *acutifolia* and yellow mosaic with brown necrotic spots on *P. obtusa*, whereas, FrMV-Ind-1 caused greenish mosaic, chocolate spots, and necrotic rings on *P. rubra* f. *acutifolia* and greenish mosaic with necrotic spots on *P. rubra* f. *obtusa* (Figure 1A)*.* Inoculation of PluMV-Plu-Ind-1 to *N. benthamiana* resulted in expression of whitish ring-spot as local symptoms and whitish wavy lines, mosaic mottling, and blistering as systemic symptoms. Whereas, *N. benthamiana* inoculated with FrMV-Ind-1 exhibited chlorotic spots as local symptoms and mosaic, mottling, and blistering as systemic symptoms (Figures 1B,C). *N. tabacum* cv. Xanthi inoculated with PluMV-Plu-Ind-1 developed whitish ring-spots and mottling as local symptoms and FrMV-Ind-1 inoculation developed necrotic white lesions as local symptoms (Figure 1D) whereas, none of the virus isolates induced any systemic symptoms in *N. tabacum* cv. Xanthi. Similarly, *N. glutinosa* developed only local symptoms with concentric whitish ring pattern when inoculated with PluMV-Plu-Ind-1, whereas, FrMV-Ind-1 induced large necrotic local lesions on *N. glutinosa* (Figure 1E). Bright red colour spots developed on all the inoculated leaves of *G. globosa,* whereas, systemic leaves were symptomless when inoculated with PluMV-Plu-Ind-1. FrMV-Ind-1 did not induce any symptoms on local as well as systemic leaves of *G. globosa* (Figure 1G). *D. stramonium* inoculated with PluMV-Plu-Ind-1 exhibited chlorotic spots on the inoculated leaves and chlorosis with mottling on the systemic leaves. However, FrMV-Ind-1 induced large blighted patches on the inoculated leaves of *D. stramonium* and no systemic symptoms (Figures 1H,I). *S. melongena* and *C. annuum* did not exhibit any symptoms when inoculated with PluMV-Plu-Ind-1, but developed chlorotic spots and chlorotic lesions, respectively upon FrMV-Ind-1 inoculation (Figures 1J,K). All the above host species showing symptoms were positive when examined by EM, ELISA and RT-PCR (Figures 1F,L), whereas, asymptomatic plants were tested negative in all the assays.

### Clones and sequence of full-length genome

The full-length genome sequence was generated cloning six overlapping fragments over the entire genome (Figure 2). In the initial attempt to amplify the genomic fragments, primers specific to cucumber green mottle mosaic virus and FrMV were used that did not amplify any fragment from the total RNA isolated either from *N. benthamiana* or *G. globosa* leaves infected with PluMV-Plu-Ind-1. Further attempt using a primer pair, BM115F (a degenerate primer for tobamovirus designed in this study from 5′ UTR region) and BM116R (FrMV specific primer) resulted in amplification of multiple bands. Sequencing of a clone containing ~1.3 kb fragment (fragment-1) from the PCR product contained part of small and large Rep gene, which showed significant differences from FrMV. Therefore, the sequence of fragment-1 was used to design a specific reverse primer (BM204R). The fragment-2 covering the 5′ region of the genome was then generated by degenerate BM115F and PluMV-Plu-Ind-1 specific BM204R. The terminal sequence was confirmed by 5′ RACE using 5′ Full RACE core set kit (Takara, Shiga, Japan), and a pair of specific primers (BM239F and BM222R) were designed based on the sequence of fragment-2 (Table 2). The fragment-2 sequence was further confirmed by generating fragment-3 using PluMV-Plu-Ind-1 specific primers, BM239F and BM222R. The fragment-4 that overlapped with fragment-1 and partly with fragment-2 was generated using PluMV-Plu-Ind-1 specific forward primer, BM348F and FrMV-Ind-1 specific reverse primer, BM240R. To generate the 3′ genome sequence, attempts were made to amplify the 3′ end genome fragment using PluMV-Plu-Ind-1 specific forward primer, BM649F designed from the sequence of fragment-4, and FrMV-Ind-1 specific reverse primers, BM119R and BM140R, unfortunately, no amplification was obtained using these primer combinations. Further attempt was made using a semi-nested PCR; where the first round of PCR was performed with BM649F and BM119R primers followed by the second round of PCR with BM649F and BM140R primers resulted in amplification of the fragment-5 containing MP and CP regions. To obtain 3′ untranslated region (UTR) sequence, the fragment-6 was generated by PluMV-Plu-Ind-1 specific BM205F primer and BM119R from FrMV-Ind-1. Further, the 3′ terminal sequence was confirmed by 3′ RACE using FirstChoice RLM RACE kit (Thermo Fischer Scientific, USA).

### Genome organization and sequence comparison of PluMV-Plu-Ind-1

**Figure 3:**
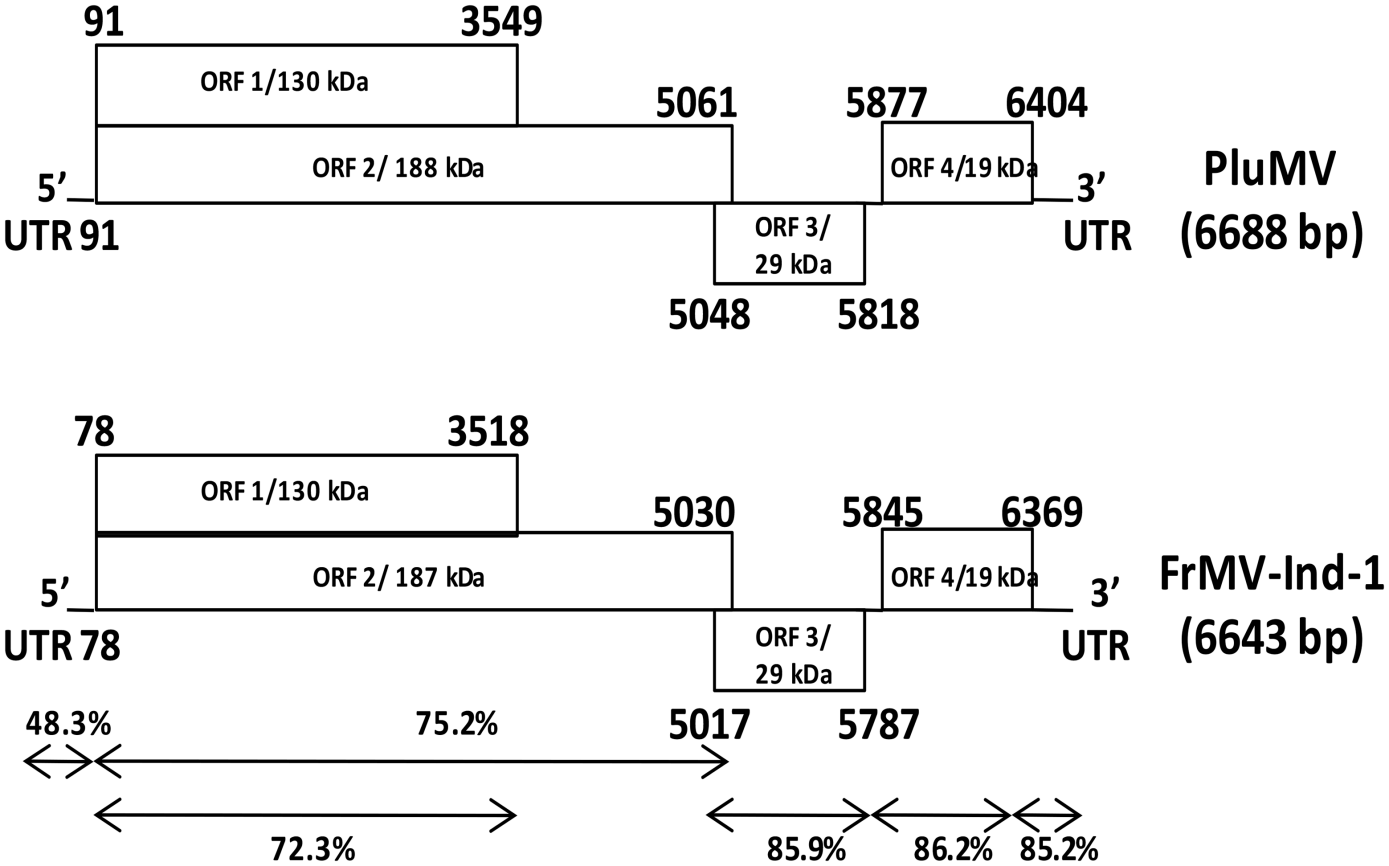
Comparison of the genome structure of plumeria mosaic virus (pluMV-Plu-Ind-1) and Indian isolate of frangipani mosaic virus (FrMV-Ind-1). The percent identities between the coding and non-coding regions of the two viruses are indicated with arrows.

The complete genome of PluMV-Plu-Ind-1 was 6,688 nts long with four ORFs (GenBank KJ395757, 2015). The sequence 1–90 nt contained 5′ UTR. The ORF1 spanned between 91 to 3,549 nt with the start codon AUG and termination codon UAG, encoding the small Rep protein of 130 kDa. The ORF2 that spanned between 91 to 5,061 nt with a readthrough leaky termination amber codon (UAG) at 3,549 nt encoded 188 kDa large replicase protein. The ORF3 (5,048–5,818 nt) that overlapped with 14 nt of the 3′ end of ORF2 encoded a 29 kDa MP. The ORF4 located 60 nt apart from the MP (5,877–6,404 nt) and encoded a 19 kDa CP. The 3′ UTR was located from 6,405 nt to the end of the genome (Figure 3).

The comparison of the sequence of PluMV-Plu-Ind-1 with the another isolate found in the database reported later from Taiwan (DR_TW; KX881422, 2018) revealed that both the isolates were almost similar to each other sharing 98% sequence identity at the genome level and 97.9–100% identity at nt and amino acids (aa) levels of non-coding and individual gene, except in 5′ UTR, which was found to be 5 nt longer than DR_TW, and shared only 91.1% nt sequence identity (Table 3). The comparison of the sequences of PluMV-Plu-Ind-1 with that of 34 other tobamovirus species showed that PluMV-Plu-Ind-1 shared 71.4–71.6% nt sequence identity with FrMV isolates and < 50% sequence identity with the rest of the other tobamovirus species (Table 3). The detailed comparison of the sequences of PluMV-Plu-Ind-1 with FrMV isolates revealed that the 3′ genomic regions (MP and CP) including the 3′ UTR of PluMV-Plu-Ind-1 shared higher sequence conservancy (>85%) with FrMV isolates compared to the 5′ genomic region including both coding (<76% with large and small Rep) and non-coding regions (48.3–49.4% with the 5′ UTR; Table 3). Interestingly, the 5′ coding region containing Rep of other tobamovirus species shared relatively higher sequence identity (34%) with PluMV-Plu-Ind-1 compared to the 3′ coding region containing MP and CP (18.8%; Table 3).

**Table 3:** Percent nucleotide/amino acid sequence identity of plumeria mosaic virus isolate, PluMV-Plu-Ind-1 from India with the isolate from Taiwan (PluMV-DR_TW), and with the other tobamoviruses.

**Virus**	Accession no.	Complete genome	5′ UTR	3′ UTR	Large Rep		Small Rep		MP		CP	
					nt	aa	nt	aa	nt	aa	nt	aa
PluMV-DR_TW	KX881422	98.0	91.1	99.2	97.9	98.7	97.7	98.6	98.3	98.8	99	100
FrMV-Ind-1	JN555602	71.4	48.3	85.2	68.8	75.2	67.1	72.3	79.5	85.9	78.7	86.2
FrMV-P	HM026454	71.6	49.4	85.5	69.1	75.3	67.3	72.2	79.5	86.3	78.5	85.7
Fr-Adel	AF165884	–	–	85.5	–	–	–	–	79.1	84.7	79.5	86.2
BrMMV	AM398436	45.4	27.1	31.0	48.6	41.1	44.2	36.1	36.7	23.9	44.6	37.1
BPMoV	DQ355023	45.2	30.4	29.2	48.2	41.6	44.4	37.0	37.3	23.7	43.6	38.9
CFMMV	AF321057	48.7	33.6	30.9	48.7	41.6	47.2	37.4	46	29.4	44.0	33.1
CGMMV	D12505	48.0	23.9	28.5	48.5	42.7	47.3	38.4	45.3	32.5	47.2	36.5
ClYMV	JN566124	44.4	30.4	34.6	48.0	42.7	44.6	39.0	33.1	21.4	44.4	36.5
CMMoV	EU043335	43.9	19.5	31.5	45.9	38.5	42.9	34.0	32.6	23.4	39.0	29.9
CuMoV	AB261167	48.8	27.1	28.3	49.0	43.4	47.8	38.9	45.1	32.4	47.8	38.8
HLFPV	FJ196834	47.2	19.5	23.5	51.1	44.1	46.9	39.9	38.3	24.5	47.8	40.0
HLSV	AF395898	46.0	23.9	26.6	49.7	44.3	47.0	41.5	38.7	24.0	46.1	39.4
KGMMV	AJ295948	48.1	32.6	24.3	48.0	42.2	46.3	38.0	44.5	29.4	43.6	34.2
MarMV	DQ356949	40.8	14.1	27.4	45.9	41.6	44.6	39.4	33.4	19.0	45.5	37.1
NTLV	AY137775	-	-	-	-	-	-	-	38.3	25.2	45.0	37.2
ObPV	D13438	43.5	27.4	41.5	47.4	41.4	42.9	36.0	38.3	23.7	40.1	37.8
ORSV	X82130	42.8	22.8	26.9	47.0	39.1	43.0	34.7	35.1	20.4	41.8	37.2
PaMMV	AB089381	44.4	29.6	33.2	47.7	41.6	43.4	36.5	37.6	25.6	43.7	37.8
PFMV	HQ389540	40.7	16.3	29.2	48.6	42.7	43.8	39.4	32.7	20.3	46.7	34.8
PMMoV	M81413	45.6	27.4	28.9	48.3	41.9	44.1	36.9	39.3	26.2	44.0	40.6
RaCNaV	JF729471	44.3	43.3	27.6	46.6	40.4	42.8	35.7	31.0	18.8	41.1	27.4
RehMV	AB628188	46.3	33.6	31.0	49.0	42.5	44.7	37.1	35.9	22.7	47.8	41.2
RMV	HQ667979	45.4	25.0	31.7	49.0	42.3	44.4	38.0	38.6	22.8	42.3	37.8
SFBV	AM040955	44.9	26.0	26.4	48.8	42.2	44.1	37.1	36.5	23.3	37.4	32.2
SHMV	MW057697	45.4	28.2	39.1	48.6	42.1	45.3	38.1	33.9	23.7	47.6	40.1
TBRFV	KT383474	45.5	34.7	32.0	48.3	42.0	44.0	36.7	36.6	22.7	47.0	41.8
TMGMV	M34077	46.1	33.6	31.3	49.5	41.4	45.0	37.4	39.8	25.7	45.1	39.5
TMV	AJ011933	45.6	32.6	31.3	48.5	42.3	43.9	37.0	35.8	21.6	45.7	41.2
ToMV	X02144	45.9	31.5	31.3	48.9	42.5	44.5	37.5	36.7	23.0	44.4	40.1
ToMMV	KF477193	45.9	33.6	29.2	49.0	42.3	44.1	37.4	39.2	22.3	45.9	40.1
TSAMV	KU659022	45.2	28.5	27.8	48.2	41.2	44.2	36.5	39.7	27.1	44.0	38.4
TVCV	U03387	45.5	25.0	30.6	49.1	42.3	44.5	37.4	38.6	21.7	43.6	38.9
WMoV	KJ207375	45.1	26.0	30.3	48.8	42.3	44.2	37.2	38.4	23.9	43.8	37.2
YoMV	AB261175	45.3	26.0	29.8	49.0	42.5	44.8	38.0	39.6	23.4	42.3	38.4
YTMMV	KF495564	45.5	31.8	25.0	49.1	41.9	44.5	36.2	37.0	24.6	41.9	34.8
ZGMMV	AJ295949	48.5	29.3	24.6	48.4	42.1	46.6	37.8	45.7	29.4	46.5	36.0

FrMV, Frangipani mosaic virus; BrMMV, Brugmansia mild mottle virus; BPMoV, Bell pepper mottle virus; CFMMV; Cucumber fruit mottle mosaic virus; CGMMV, Cucumber green mottle mosaic virus; ClYMV, Clitoria yellow mottle virus; CMMoV, Cactus mild mottle virus; CuMoV, Cucumber mottle virus; HLFPV, Hibiscus latent fort pierce virus; HLSV, Hibiscus latent Singapur virus; KGMMV, Kyuri green mottle mosaic virus; MarMV, Maracuja mosaic virus; NTLV, Nigerian tobacco latent virus; ObPV, Obuda pepper virus; ORSV, Odontoglossum ringspot virus; PaMMV, Paprika mild mottle virus; PFMV, Passion fruit mosaic virus; PMMoV, Pepper mild mottle virus; RaCNaV, Rattail cactus necrosis-associated virus; RehMV, Rehmannia mosaic virus; RMV, Ribgrass mosaic virus; SFBV, Streptocarpus flower break virus; SHMV, Sunn-hemp mosaic virus; TBRFB, Tomato brown rugose fruit virus; TMGMV, Tobacco mild green mosaic virus; TMV, Tobacco mosaic virus; ToMV, Tomato mosaic virus; ToMMV, Tomato mottle mosaic virus; TSAMV, Tropical soda apple mosaic virus; TVCV, Turnip vein clearing virus; WMoV, Wasabi mottle virus; YoMV, Youcai mosaic virus; YTMMV, Yellow tailflower mild mottle virus; ZGMMV, Zucchini green mottle mosaic virus; −, not available; UTR, Untranslated region; Rep, Replicase; MP, Movement protein; CP, Coat protein

### Comparison of genome organization of PluMV with that of FrMV

**Figure 4:**
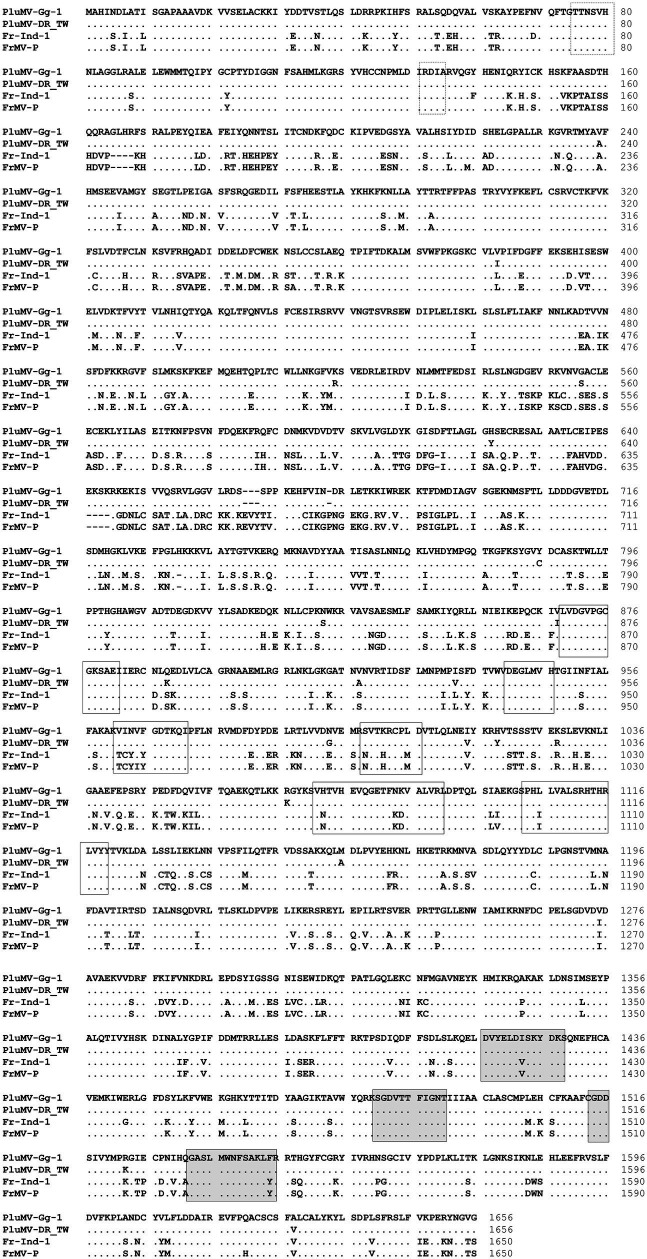
Comparison of the amino acid sequence of the large replicase protein of two isolates of plumeria mosaic virus (PluMV-Plu-Ind-1 and PluMV-DR_TW) with that of the isolates of closely related frangipani mosaic virus (FrMV-Ind-1 and FrMV-P). Boxes with dotted line showing conserved motifs of methyltransferase domain, boxes with continuous line showing motifs for helicase domain, and shaded boxes showing motifs for polymerase domain. Dot indicates identical amino acid, dash indicates gap.

The comparison of PluMV sequence with that of FrMV-Ind-1 (JN555602) and FrMV-P (HM026454) revealed that the complete genome of PluMV was 45 nt longer than both the isolates of FrMV. The 5′ and 3′ UTRs of PluMV was 13 nt and 10 nt longer, respectively than that of FrMV. The 5′ UTR was relatively more diverse (48.3–49.4% sequence identity) than the 3′ UTR (85.2–85.5% sequence identity; Table 3; Figure 3). The proximal 3′ nts of PluMV contained GTCCCC, which is different from both the FrMV and most of the tobamovirus isolates (data not shown). The MP and CP of PluMV shared closer amino acid (aa) sequence identity (84.7–86.3%) compared to both the Rep (72.2–75.3%) with that of FrMV. Both the Rep proteins of PluMV were 9 aa longer than FrMV isolates and shared 72.2–72.3% and 75.2–75.3% aa sequence identity, respectively. MP of PluMV was of identical in length sharing 84.7–86.3% aa sequence identity, whereas, CP of PluMV was one aa longer and shared 85.7–86.2% identity with FrMV isolates (Table 3). The arrangement of MP and CP genes in genome for both the viruses was very similar. The MP of both the virus overlapped with the terminal 13 nt of large Rep. But, the length of the intergenic region between MP and CP was only one nt longer for PluMV (Figure 3). The comparison of aa sequence of 130 kDa and 188 kDa Rep proteins showed that the aa sequence was highly conserved (only 19 substitutions) within the PluMV isolates from India and Taiwan (PluMV-Plu-Ind-1 and DR_TW), but highly diverse between PluMV and FrMV. The major dissimilarities in Rep protein was found in three stretches, i.e., from 153–164, 646–669, and 674–697 aa positions in the form of substitutions as well as deletions (Figure 4). Apart from these, region corresponds to 182–190, 335–352, and 546–563 positions have also weak aa identity (Figure 4). Irrespective of these many of dissimilarities in 130 kDa/188 kDa proteins of PluMV and FrMV, it was well orchestrated with all three conserved domains; methyltransferase, helicase, and polymerase. The methyltransferase domain (235–1,419 nt) of PluMV, responsible for capping of genomic and sub-genomic RNAs was 12 nt longer than FrMV whereas, the helicase domain (2,692–3,453 nt) responsible for unwinding of nucleic acid, recombination, and transcription (Alonso et al., 1991), and polymerase domain (3,700–5,025 nt) responsible for the elongation of pre-existing chains (Quadt and Jaspars, 1989) were of similar in length. The two conserved sequence motifs (invariant His in the first motif and Asp-X-X-Arg signature site in the second motif) of methyltransferase as described by Rozanov et al. (1990) were well conserved in both the viruses (Figure 4). Among the six conserved sequence motifs for the helicase domain of tobamovirus (Goldbach and Wellink, 1988), motif I and II were well conserved for both the viruses whereas, some conservative and non-conservative substitutions were seen in motif III to VI of PluMV in comparison to FrMV (Figure 4). The nucleotide sequences (ATAGCAATTACAG) at the position of termination of 130 kDa protein are strictly conserved in all the tobamoviruses (Strauss et al., 1988), but in case of both PluMV and FrMV, the last three nt sequences were not conserved, and were replaced by ATG, whereas in PluMV-DR_TW, it was replaced by GCG (data not shown). The four conserved sequence motifs (A - D) of polymerase (Poch et al., 1989) were also conserved for both the viruses except a single substitution of Val to Ile at position 1,423 in motif-A and Tyr to Phe at position 1,545 in motif-D in FrMV isolates (Figure 4). In tobamovirus Rep protein, the consensus sequences (GXXXXGKT and DEAD box in the helicase domain, and GDD and SGXXXTXXXNT in polymerase domain) were also conserved, except the last amino acid Thr in GXXXXGKT has been substituted by Ser in both the viruses (Figure 4).

### Phylogenetic relationships

**Figure 5:**
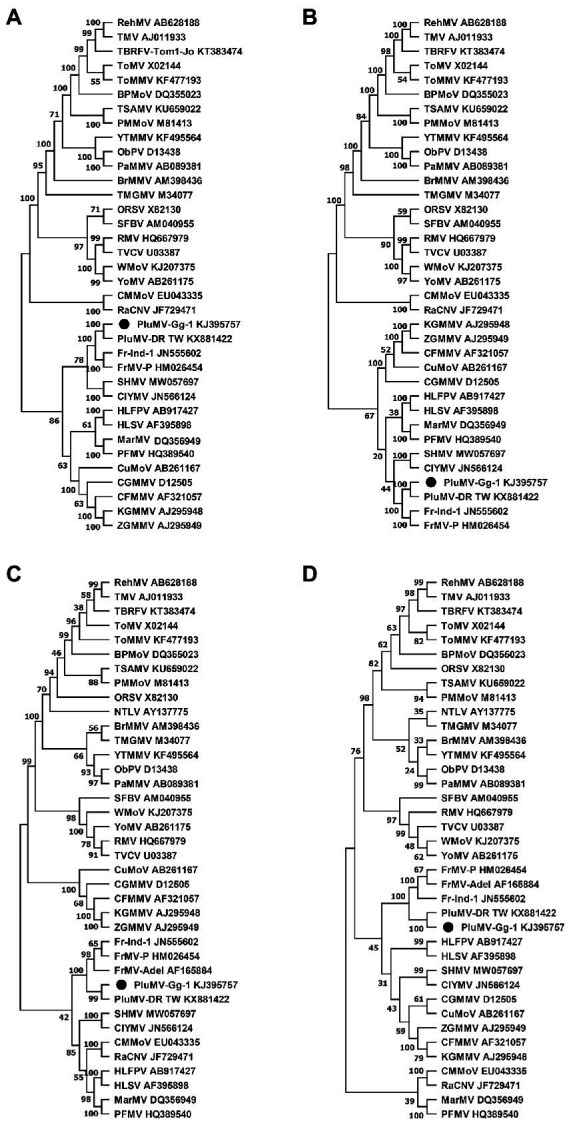
Phylogenetic tree of plumeria mosaic virus isolate, PluMV-Plu-Ind-1 and members of the genus *Tobamovirus* based on the 188 kDa large replicase protein **(A)**, the 130 kDa small replicase protein **(B)**, the 30 kDa movement protein **(C)**, and the 18 kDa coat protein **(D)**. The tree was constructed based on maximum likelihood method using MEGA version 11 with 1,000 bootstraps value. Full name of the viruses is indicated in Table 3.

Phylogenetic analyses based on the amino acid sequences of each protein showed that PluMV was closely related to FrMV, however PluMV formed a separate branch away from FrMV isolates (Figure 5). The phylogenetic analysis of both the Rep proteins revealed that PluMV together with FrMV was closely related with clitoria yellow mottle virus and sunn-hemp mosaic virus (Figures 5A,B), and to the lesser extent to malvaceae-, passifloraceae- and cucurbitaceae-infecting tobamoviruses. Phylogenetic analysis of MP indicated that both the PluMV and FrMV isolates were somewhat related with cucurbitaceae-, brassicaceae-and solanaceae-infecting tobamoviruses (Figure 5C), whereas, the CP of PluMV and FrMV were more related with solanaceae- and brassicaceae-infecting tobamoviruses (Figure 5D).

### Detection of PluMV and FrMV in naturally infected temple tree plants

**Figure 6:**
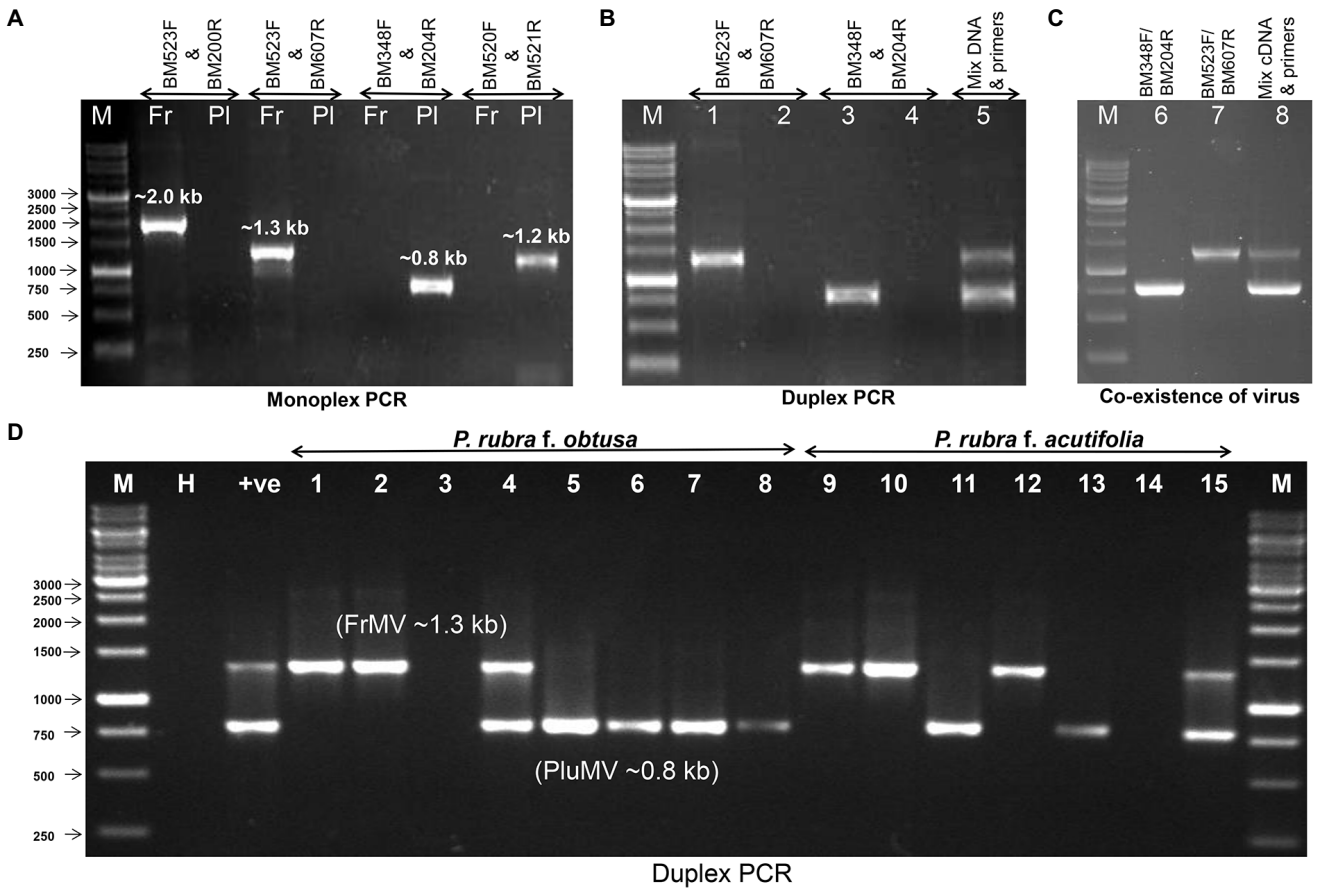
Detection of frangipani mosaic virus (FrMV-Ind-1) and plumeria mosaic virus (PluMV-Plu-Ind-1) by RT-PCR. **(A)** Optimization of specificity of the primers using the cloned DNA of FrMV-Ind-1 and PluMV-Plu-Ind-1. **(B)** Duplex PCR using the cloned DNA of FrMV-Ind-1 and PluMV-Plu-Ind-1 with the specific primers BM523F/BM607R and BM348F/BM204R, respectively. M: Marker, Lane 1: FrMV clone tested by FrMV specific primers, Lane 2: FrMV clone tested by PluMV specific primers, Lane 3: PluMV clone tested by PluMV specific primers, Lane 4: PluMV clone tested with FrMV specific primers, Lane 5: Duplex PCR (mixture of both the clone tested with mixture of primers). **(C)** Confirmation of co-infection of both the virus (FrMV and PluMV) in original frangipani tree (*Plumeria rubra* f. *acutifolia*) from where both the viruses were isolated. M: Marker, Lane 1: RT-PCR by FrMV specific primers, Lane 2: RT-PCR by PluMV specific primers, Lane 3: Duplex RT-PCR with the mixture of both the primers. **(D)** Duplex RT-PCR confirmation of single and mixed infection of PluMV and FrMV in the leaf samples collected from different trees at IARI campus. M: Marker, H: Healthy, +ve: Duplex RT-PCR from RNA extracted from the original frangipani tree, Lane 1–15: leaf samples. Fr: FrMV; Pl: PluMV.

The antiserum developed using purified virus preparation of FrMV-Ind-1 (Kumar et al., 2015) was used for primary detection of both the viruses in ELISA (data not shown). Further, two pairs of specific primers BM348F/BM204R and BM520F/BM521R were optimized for the specific detection of PluMV, which resulted in amplification of ~0.8 kb from fragment-4 and ~ 1.2 kb from fragment-3 of PluMV cloned DNA (Figure 2), respectively in RT-PCR, whereas, no amplification was found with the FrMV-Ind-1 cloned DNA. Similarly, BM523F/BM200R and BM523F/BM607R have been optimized for the specific detection of FrMV-Ind-1, which resulted in amplification of ~2.0 kb and ~ 1.3 kb bands in the RT-PCR, respectively only with the FrMV-Ind-1 cloned DNA and not with the PluMV cloned DNA (Figure 6A). The primer pairs BM348F/BM204R and BM523F/BM607R were then used to detect the virus in the inoculated plants, where all the symptomatic plants inoculated with PluMV-Plu-Ind-1 and FrMV-Ind-1 gave a specific amplification of ~0.8 kb and ~ 1.3 kb amplifications, respectively in RT-PCR (Figure 1L), whereas, non-symptomatic plants did not give any amplification. Further, a duplex PCR system has been established for the simultaneous detection of PluMV and FrMV using the mixture of both the primer sets (BM348F/BM204R and BM523F/BM607R) and cloned DNA of PluMV-Plu-Ind-1 and FrMV-Ind-1 (Figure 6B). The simplex and duplex PCR systems were successfully utilized to confirm the presence of both the viruses in the original temple tree from where the virus was originally isolated. A specific amplification of ~0.8 kb band for PluMV-Plu-Ind-1 and ~ 1.3 kb band for FrMV-Ind-1 was obtained in simplex RT-PCR performed with the RNA extracted from the symptomatic leaf collected from the original temple tree and two bands of the desired size were obtained in the duplex RT-PCR with the same RNA indicating mixed infection of both the viruses (Figure 6C). The RT-PCR testing of leaf samples of temple trees from the different locations at IARI campus revealed that FrMV was common in *P. rubra* f. *acutifolia* as 4 out of 7 leaf samples of acutifolia trees were positive for FrMV infection; whereas, PluMV was found common in *P. rubra* f. *obtusa* as 5 out of 8 leaf samples of obtusa trees were positive for PluMV infection. However, both the *Plumeria* species were found susceptible for both the viruses, as single and mixed infections were detected in both the plant species using duplex RT-PCR (Figure 6D)*.*

## Discussion

Temple tree was known to be infected with a tobamovirus, FrMV (Lim et al., 2010; Kumar et al., 2015). In this study, we described another tobamovirus, PluMV infecting temple tree in India. The PluMV was easily sap-transmissible to temple tree plants, *P. rubra* f. *acutifolia* and *P. rubra* f. *obtusa,* and also to many herbaceous plant such as datura, globe amaranth and tobacco*.* While conducting host-range study, we observed that the symptom development due to PluMV and FrMV infection was influenced by temperature (unpublished observation), as no symptoms were observed in most of these plant species below 27°C, except *N. benthamiana*, which produced mottling mosaic symptoms below 27°C, but took long incubation period for the expression of symptoms. The temperature even influenced the expression of a particular type of symptoms, for example, the inoculated *N. benthamiana* plants did not develop the whitish ring-spot and wavy whitish lines symptoms at a temperature below 27°C, but the same appeared at 30–35°C. *N. benthamiana* appears to be a suitable host for the maintenance of PluMV. Previously, similar influence of temperature on host range and symptomatology was also documented in case of FrMV infecting temple tree (Varma and Gibbs, 1978).

The comparative transmission studies of both the viruses in various plant species helped in identifying three plant species, *G. globosa, S. melongena* and *C. annuum* as important differential plant species of PluMV and FrMV. The red local spot in *G. globosa* is a diagnostic symptom of PluMV. Although, the systemic symptom developed by PluMV on *D. stramonium* could also be used as differentiating symptoms of both the viruses. The differential symptomatology observed in a particular plant species under the similar growing conditions is expected due to the difference in the genetic makeup of the two viruses, PluMV and FrMV.

The complete genome sequence revealed that PluMV had a genome structure typical to the genus, *Tobamovirus* (Adams et al., 2017). Like the subgroup-1 tobamoviruses, the MP and CP genes of PluMV were arranged in the genome without overlapping with each other. Another property of the subgroup-I tobamoviruses is that they generally infect solanaceous plant species. PluMV was originally isolated from *P. rubra* f. *acutifolia* of the family Apocynaceae. Our study showed that PluMV also infected solanaceous plant species such as *D. stramonium, N. benthamiana, N. glutinosa* and *N. tabacum.* Based on the feature of genome architecture and host biology, PluMV could be considered as a new member of the subgroup-I tobamoviruses. The genome sequence comparison of the members of the genus, *Tobamovirus* showed that PluMV was most closely related to FrMV, with only 71.4–71.6% nucleotide sequence identity. For considering a new tobamovirus species, the ICTV guideline is that the complete genome sequence of the candidate member should have less than 90% sequence identity with the recognised members (Adams et al., 2017). Further, based on the distinct phylogenetic relationships, PluMV was considered as a new tobamovirus species. The comparison of the amino acid sequences of large Rep protein of PluMV with that of FrMV showed that, irrespective of significant dis-similarities in the sequences, the major domains (methyltransferase, helicase and polymerase) of the tobamovirus were well conserved in PluMV, except, few substitutions in the helicase and polymerase domains.

Host-range study showed that temple tree was the common host of PluMV and FrMV. Both the viruses could not be readily differentiated visually by symptomatology alone on temple tree. The antiserum developed to FrMV was unable to differentiate both the viruses. Additionally, the CP based primers, designed for the detection of FrMV-Ind-1 (Kumar et al., 2015) also could not detect PluMV. The difference in the genome sequence of both the virus was utilized to develop a specific RT-PCR test for each of these viruses infecting temple tree. The primer pairs developed in this study from the most dissimilar region in the Rep gene successfully differentiated both the viruses by simplex as well as duplex RT-PCR. The RT-PCR diagnosis revealed the natural existence of PluMV alone or together with FrMV. In 2018, 3 years after our submission of the genome sequence in the NCBI database, PluMV was also found in Taiwan infecting desert rose plant (*Adenium obesum*), another ornamental plant of the family Apocynaceae (GenBank KX881422). Detection of PluMV independently in the different trees of both the species of temple tree (*P. rubra* f. *acutifolia* and *P. rubra* f. *obtusa*) in India as well as in *A. obesum* in Taiwan, provided evidence of natural existence of the new tobamovirus.

FrMV has been detected in temple tree in the different countries (Lim et al., 2010; Kumar et al., 2015; Choliq et al., 2017; Dey et al., 2020), whereas, PluMV is yet to be documented in the various parts of the World. Temple tree is commercially propagated through stem cutting. Tobamoviruses being highly contagious, both FrMV and PluMV in temple tree can easily be circulated through cuttings. The comparative host biology and molecular diagnosis presented in this study will be useful in production of virus free planting materials of temple tree. As these viruses co-infect temple tree, synergistic or antagonistic effect on the performance of temple tree needs to be investigated in further study.

## Data availability statement

The datasets presented in this study can be found in online repositories. The names of the repository/repositories and accession number(s) can be found below: https://www.ncbi.nlm.nih.gov/genbank/, KJ395757.

## Author contributions

AK conceptualized, designed, performed, executed the research experiments and written the manuscript. VS and AKat assisted in experimentation. BM conceptualized, guided the research experiments, and corrected the manuscript. All authors contributed to the article and approved the submitted version.

## Conflict of interest

The authors declare that the research was conducted in the absence of any commercial or financial relationships that could be construed as a potential conflict of interest.

## Publisher’s note

All claims expressed in this article are solely those of the authors and do not necessarily represent those of their affiliated organizations, or those of the publisher, the editors and the reviewers. Any product that may be evaluated in this article, or claim that may be made by its manufacturer, is not guaranteed or endorsed by the publisher.
